# A Case of Giant Neobladder Stone of 5 Kg

**DOI:** 10.1002/iju5.70026

**Published:** 2025-04-09

**Authors:** Kaede Hiruma, Masayuki Tasaki, Tsutomu Anraku, Masahiro Ikeda, Kazuhide Saito, Yoshifumi Shimada, Yoshihiko Tomita

**Affiliations:** ^1^ Division of Urology, Department of Urology Niigata University Graduate School of Medical and Dental Sciences Niigata Japan; ^2^ Division of Digestive and General Surgery Niigata University Graduate School of Medical and Dental Sciences Niigata Japan

**Keywords:** bladder cancer, bladder stones, cystectomy, neobladder stone, urinary stone

## Abstract

**Introduction:**

Radical cystectomy is the standard treatment for muscle‐invasive bladder cancer. A neobladder is created as part of urinary diversion; however, the formation of bladder stones is often a late complication‐related concern.

**Case Presentation:**

A 56‐year‐old man underwent radical cystectomy and neobladder construction for bladder cancer 25 years ago. A huge neobladder stone was diagnosed when the patient developed a strangulated ileus. Open surgery was performed to remove the bladder stone. The stone's size was 21.6 × 15.5 × 18.5 cm and its weight was 5 kg.

**Conclusion:**

To the authors' knowledge, this is the largest case of bladder calculus reported to date.

**Summary**

- When encountering a large new bladder stone, it is important to consider treatment methods and the prevention of recurrence.

## 1 Introduction

The first‐line treatment for muscle‐invasive bladder cancer is radical cystectomy with urinary diversion. The neobladder has the significant advantage of not requiring a stoma among the urinary diversion options. However, one of the major late complications associated with a neobladder is stone formation. About 0.5%–7% of patients who have undergone spontaneous voiding neobladder surgery developed neobladder stones [1, 2, 3]. In patients who received neobladder augmentation using the Studer technique, neobladder stones are typically detected between 61 and 132 months after surgery, with a median of 99 months [2]. The most common types of stones are calcium phosphate stones and struvite calculi [4, 5, 6]. This study summarized the reports documenting relatively large neobladder stones (Table 1). In the case of the original bladder, the stone reported by Chengquan Ma, measuring 13.3 × 8.0 × 9.7 cm and weighing 1048 g, is considered the largest [7].

**TABLE 1:** Previously reported giant neobladder stones.

No.	Age	Size (cm)	Weight (g)	Author	Year
1	67	8 × 7.5	108	Yuji Hatanaka	2008
2	60	12	860	Albert Tiu	2014
3	64	12 × 9.5 × 7.5	770	Sabine Nguyen	2017
4	68	9 × 9	—	Dongming Lu	2022
5	70	13 × 11.5 × 9	903	Jun Gu	2023
6	56	21.6 × 15.5 × 18.5	5000	Author	2025

## 2 Case Report

**FIGURE 1:**
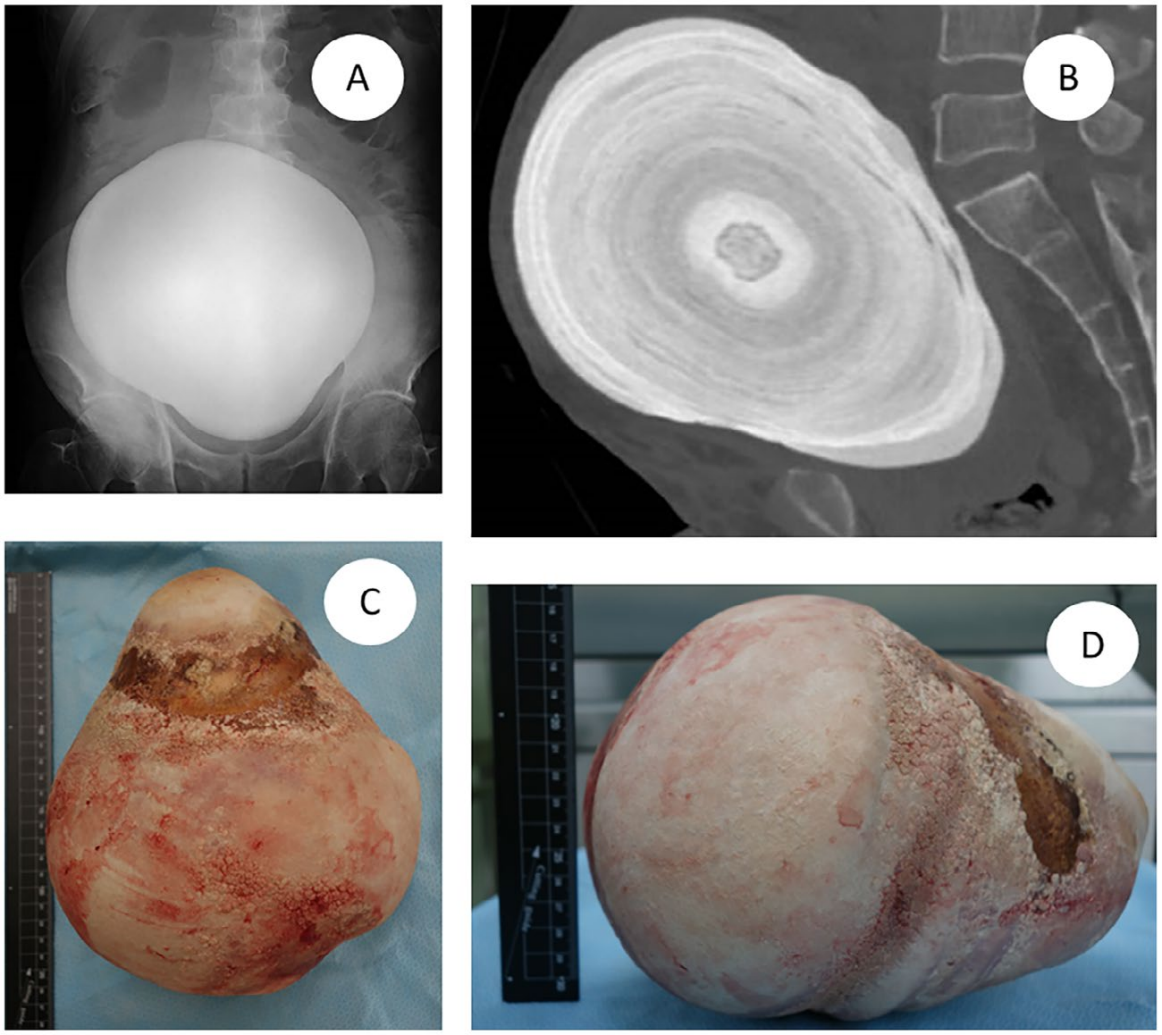
Neobladder stone in this case. (A) Abdominal X‐ray image. (B) Sagittal view of plain CT scans. (C, D) The removed stone.

**FIGURE 2:**
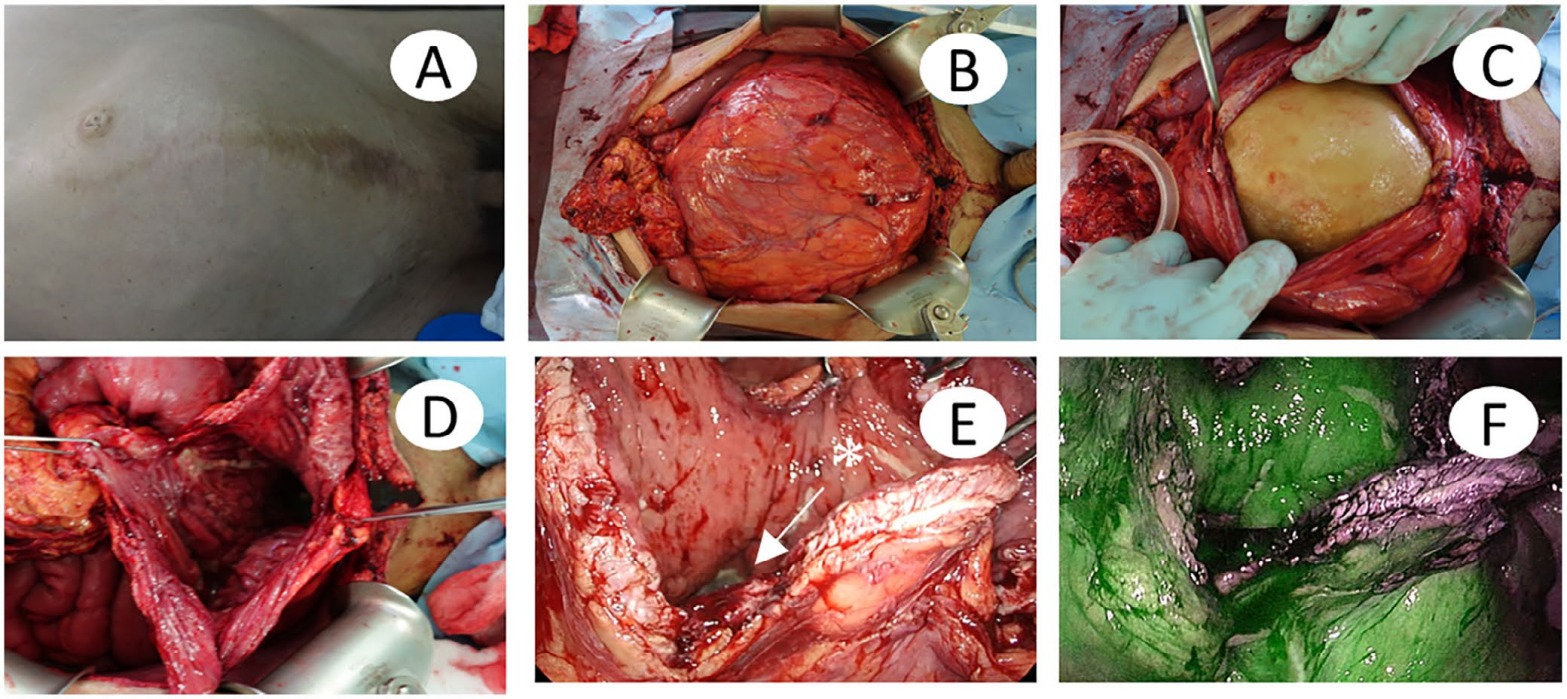
Intraoperative photographs. (A) There is a scar from a midline incision on the lower abdomen. (B–D) A longitudinal incision was made in the expanded neobladder, and the stone was removed. (E) The balloon of the indwelling catheter could be seen inside the new bladder (*). (F) After administering ICG intravenously and irradiating it with NIR light, the areas with blood flow glowed green.

A 56‐year‐old man, an automobile mechanic, visited a local clinic because of abdominal pain. He underwent radical cystectomy and neobladder augmentation surgery for muscle‐invasive bladder cancer > 25 years ago. He had no comorbidities, was not on any medication, and was unaware of urination‐related symptoms. He stopped attending medical checkups a few years after the surgery because his work schedule became hectic. His medical records from the hospital were unavailable. However, they were obtained from the physician involved in the surgery, confirming that the neobladder with an afferent limb was constructed using a colonic–ileal segment. The patient was aware of a firm abdominal mass for several years; however, because there were no troublesome symptoms, he had been monitoring it on his judgment. We detected distension in his abdomen and palpated a movable, stone‐like hard mass. He did not experience pain at the site of the mass but complained of pain above it. X‐ray (Figure 1A) and computed tomography (CT) (Figure 1B) revealed a gigantic stone measuring 21.6 × 15.5 × 18.5 cm in his neobladder and signs of strangulated ileus. The CT scan also revealed advanced left and mild right hydronephrosis, with a slight increase in creatinine. We hypothesized that this was due to dehydration, and creatinine was normalized before surgery. Moreover, the HCO_3_^−^ level was within the normal range. A partial enterectomy was performed for the strangulated ileus and open lithotripsy for the neobladder stone (Figure 1C,D). The operation time was 3 h and 22 min, with a blood loss of 30 mL. The patient had a slender physique, making it easy to observe the abdominal distension caused by the stone (Figure 2A), and the stone weighed 5 kg. When the neobladder was opened by the middle incision, no adhesion was found between the stone and the neobladder mucosa (Figure 2B–D). There was a concern about the mucosal blood flow impairment of the neobladder caused by a large calculus over an extended period. Therefore, blood flow was assessed using indocyanine green (ICG) and near‐infrared (NIR) light, confirming sufficient blood flow to the neobladder (Figure 2E,F). After surgery, he needed to perform self‐catheterization and bladder irrigation due to residual urine and to remove intestinal mucus. The stone analysis showed 79% MgNH_4_PO_4_ and 21% Ca_3_(PO_4_)_2_. Blood tests showed a pH of 7.43, corrected calcium of 10.1 mg/dL, 25‐hydroxyvitamin D of 14.70 ng/mL, intact parathyroid hormone (PTH) of 57.0 pg/mL, and PTH‐related protein < 1.0 pmol/L. Urine tests indicated a pH of 6.5–7.0 and calcium excretion of 56 mg/day. Urine cultures detected *Enterobacter cloacae* , *Staphylococcus aureus* , *Gemella haemolysans* , *Veillonella* sp., Gram‐positive rod, and α‐*Streptococcus*. He has been free of recurrent stones for 1 year.

## 3 Discussion

**FIGURE 3:**
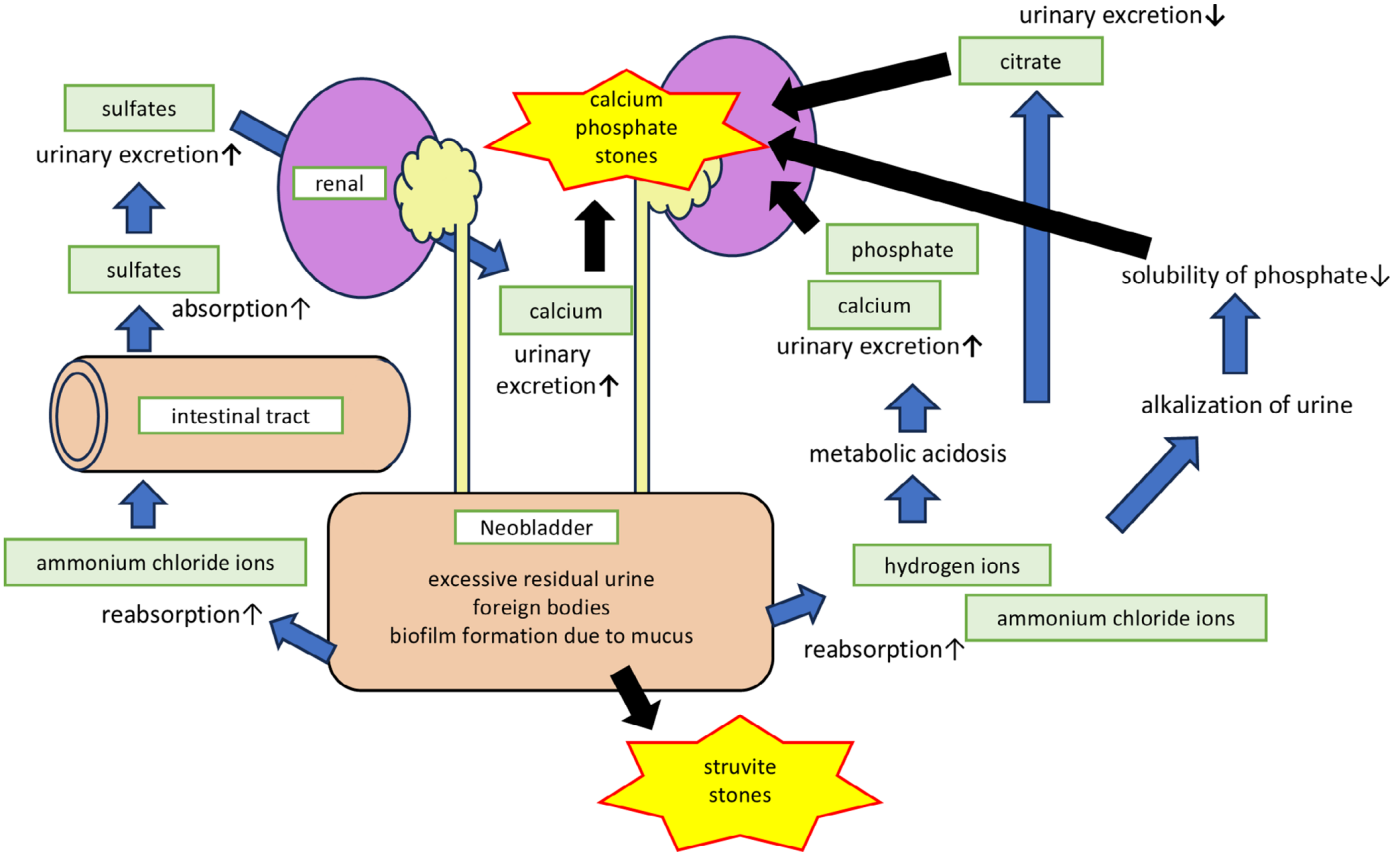
Mechanism of the formation of neobladder stones.

There are several pathways for the formation of neobladder stones. One of these involves reabsorbing hydrogen ions and ammonium chloride ions from the intestinal mucosa. This reabsorption can lead to intensified metabolic acidosis in the blood, promoting the urinary excretion of calcium and phosphate while decreasing the urinary excretion of citrate. As a result, the risk of calcium phosphate stone formation increases [1, 8]. In contrast, urine remaining in the neobladder becomes increasingly alkaline, which decreases phosphate solubility and promotes the precipitation of calcium phosphate stones within the bladder [9]. This suggests that checking for the presence of acidosis during blood tests or alkalization of urine after radical cystectomy may help prevent stone formation. The reabsorption of ammonium chloride also facilitates sulfate absorption from the intestinal tract, which aids in the urinary excretion of sulfates and promotes calcium release into the urine [1]. Another contributing factor is the state of urinary retention caused by voiding dysfunction, which can lead to excessive residual urine. The capacity of the neobladder is reported to be ~400–500 mL at 6 months postoperatively, with ~3.5% of patients eventually gaining > 1000 mL [10]. It has also been indicated that the position of the urethra formed in the neobladder may influence the risk of dysuria [11]. Ultrasound measurements of residual urine can be useful for assessing residual urine and checking for existing stones. Therefore, it may be beneficial to occasionally incorporate ultrasound examinations. Other factors include biofilm formation from foreign bodies in the bladder, such as staples and mucus, which have also been reported as causes of stone formation [9]. These pathways leading to stone formation are illustrated in Figure 3.

Giant bladder stones are those weighing > 100 g or measuring > 4 cm, and their treatment often involves open surgery [10, 12]. There are reports of strong adhesion between the stone and the neobladder mucosa as well as the neobladder‐enteric fistulas due to stones, making it crucial to consider that the neobladder has limited elasticity and lacks a muscular layer compared to normal bladders when making treatment decisions [4, 13]. During this operation, there were concerns about the lack of blood flow to the neobladder due to distension and possible adhesions between the stone and the bladder wall. Therefore, ICG was used to confirm blood flow to the neobladder, which allowed us to perform the treatment safely. Although bladder reduction surgery might be considered, the course of blood flow could not be determined. Consequently, we decided not to perform bladder reduction surgery in the current case.

To prevent the formation of neobladder stones, regular self‐catheterization and bladder irrigation are recommended, and we continue to perform regular bladder irrigation in accordance with this guidance. It has also been suggested that drinking > 2000 mL water daily may help prevent stone recurrence [11]. Some reports suggest that the environment conducive to struvite stone formation includes a urinary pH > 7.2 and an increase in urinary ammonia levels. Additionally, the solubility of struvite stones increases when pH drops below 6.5 [14]. Furthermore, the oral intake of citrate preparations could prevent acidosis and improve hypocitraturia [8]. In the current case, there was no evidence of acidosis or alkaline urine, and the urinary calcium levels were normal. It is believed that voiding dysfunction and biofilm formation due to the intestinal mucus were the primary causes. Therefore, it was determined that regular catheterization and bladder irrigation are the most effective measures for preventing neobladder stones.

## 4 Conclusion

We summarized the key considerations for treating large neobladder stones as well as the factors contributing to their formation.

## Conflicts of Interest

The authors declare no conflicts of interest.
